# High-flow nasal oxygen as an adjunct for the safe removal of impacted metallic upper airway foreign bodies

**DOI:** 10.1093/jscr/rjad147

**Published:** 2023-03-20

**Authors:** Nadia van den Berg, Moustafa Aly, Michael Callaghan, Ivan Keogh

**Affiliations:** Department of Otolaryngology Head & Neck Surgery, Galway University Hospital, Galway, Ireland; Department of Otolaryngology Head & Neck Surgery, Galway University Hospital, Galway, Ireland; Department of Anaesthetics, Galway University Hospital, Galway, Ireland; Department of Otolaryngology Head & Neck Surgery, Galway University Hospital, Galway, Ireland; School of Medicine, National University of Ireland Galway, Galway, Ireland

**Keywords:** apnoeic oxygenation, high-flow nasal oxygen, upper airway foreign body

## Abstract

Foreign body airway obstruction is considered an airway emergency and is a challenging clinical scenario for both the otolaryngologist and the anaesthetist. We present three cases of impacted upper airway metallic foreign bodies. Supra-glottic airways were obstructed and precarious. Apnoeic oxygenation utilizing high-flow nasal oxygen (HFNO), a form of tubeless anaesthetic, was used in all three cases, leading to the safe removal of the foreign bodies. Increased training, awareness and equipment availability to provide HFNO apnoeic oxygenation in the emergency setting for otolaryngology airway procedures will lead to better outcomes for patients and decreases the risk of a potential surgical airway.

## INTRODUCTION

Metallic foreign body airway obstruction is considered an airway emergency and is a challenging clinical scenario for both the otolaryngologist and the anaesthetist, particularly when these cases present in children. Most cases of upper airway foreign bodies occur in children under the age of five [1–2], with an associated mortality risk of 2.75–3.70% [3]. In certain cases, intubation may not be possible due to airway obstruction by the foreign body, or risk of further dislodgement of the foreign body. Apnoeic oxygenation utilizing high-flow nasal oxygen (HFNO) is a form of tubeless anaesthetic, which has recently gained popularity in otolaryngology, and specifically microlaryngoscopy procedures [4].

## CASE SERIES

We present three emergency cases in which metallic foreign bodies in the upper aerodigestive tract prevented endotracheal intubation. Apnoeic oxygenation using high-flow nasal oxygenation was used as a safe alternative in all three cases.

**Figure 1:**
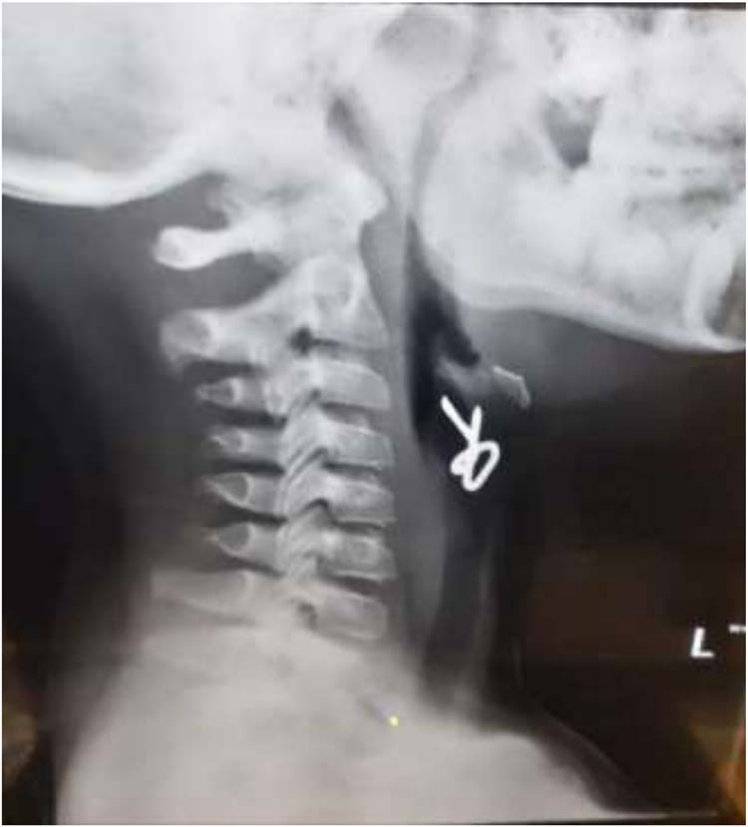
Lateral neck X-ray showing a radio-opaque object with sharp edges visible.

**Figure 2:**
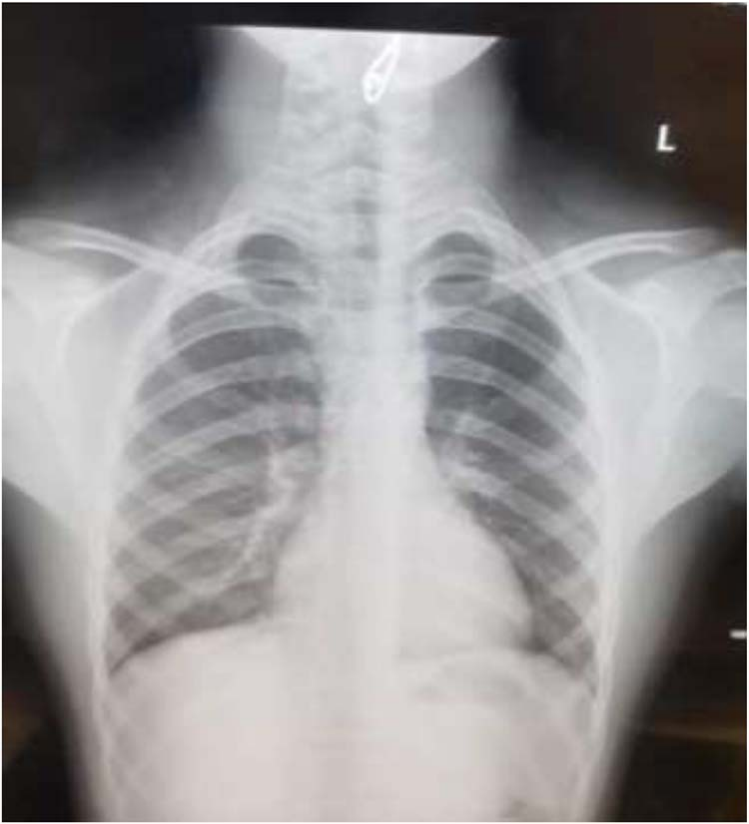
AP chest radiograph showing the sharp-edged radio-opaque object.

**Figure 3:**
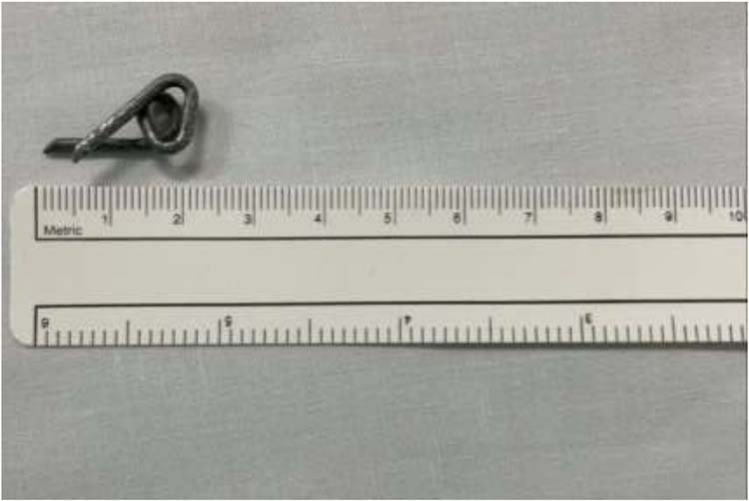
Successful removal of the barbed wire measuring ⁓2.0 cm.

The first case is of a 7-year-old child presenting with a history of barbed wire inhalation. X-ray imaging of his neck and chest showed a radio-opaque object visible in the upper airway with sharp edges (Figs 1 and 2). Due to the position of the foreign body, endotracheal intubation was not feasible. A trial of apnoeic oxygenation using high-flow nasal oxygenation was used as an alternative, and this allowed for an unobstructed view of the larynx, facilitating the safe removal of the foreign body (Fig. 3).

**Figure 4:**
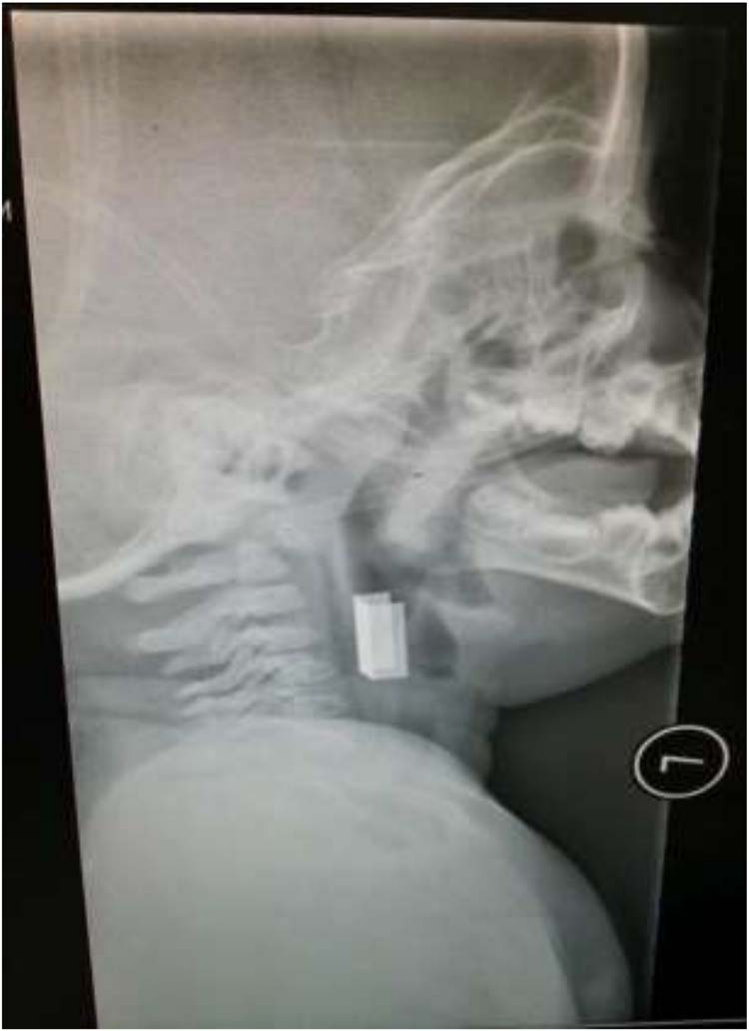
Lateral neck X-ray showing a radio-opaque object in the upper airway.

**Figure 5:**
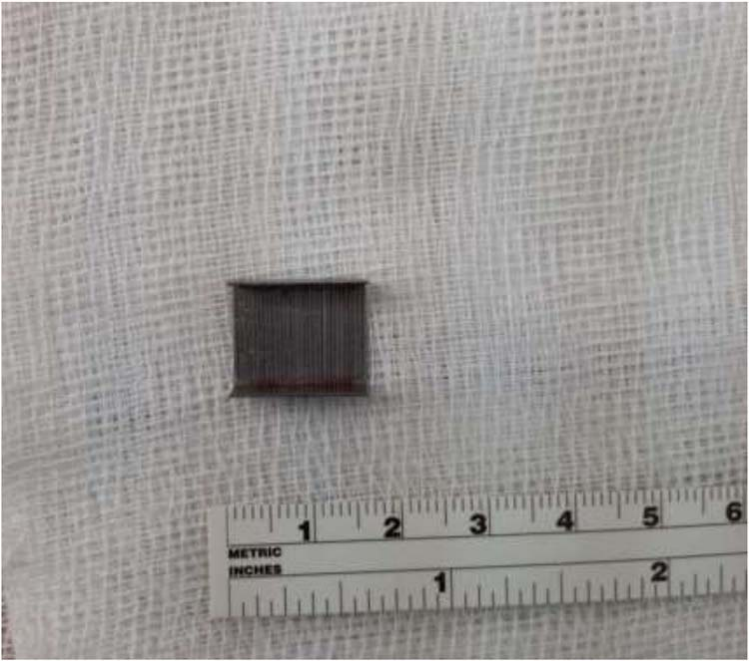
Successful removal of staple block from the upper airway.

The second case involved a 3-year-old child who presented with sudden onset noisy breathing. Inspiratory stridor was evident on examination. Radiographs of the neck again showed a radio-opaque object in the upper airway (Fig. 4). Apnoeic oxygenation allowed for tubeless anaesthetic and safe removal of the metallic foreign body from the airway (Fig. 5).

**Figure 6:**
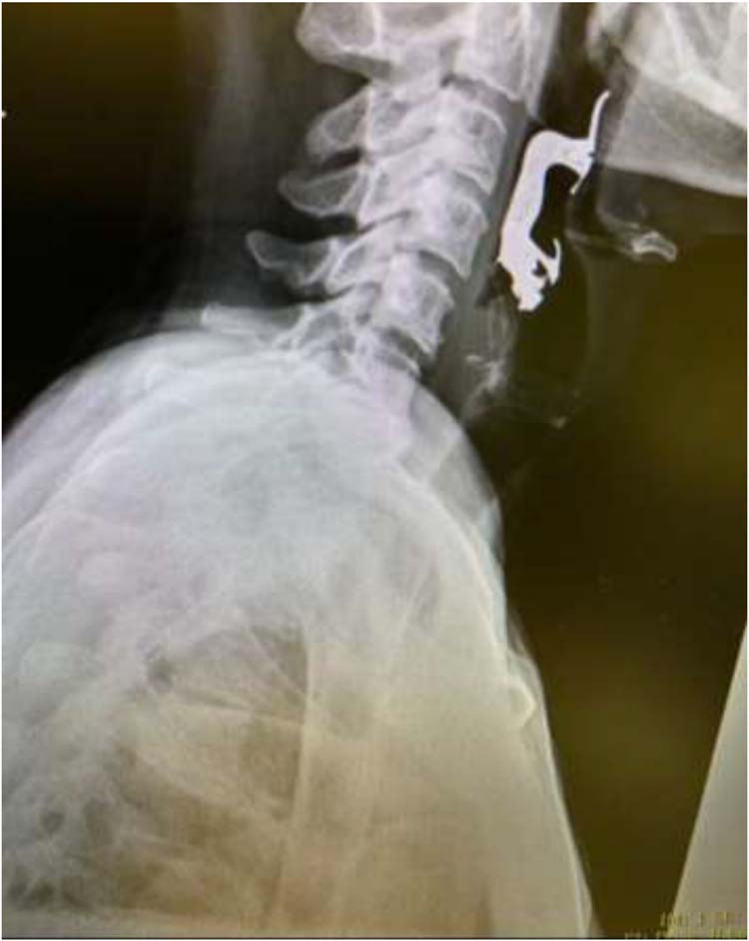
Lateral neck X-ray showing radio-opaque object in the upper airway.

**Figure 7:**
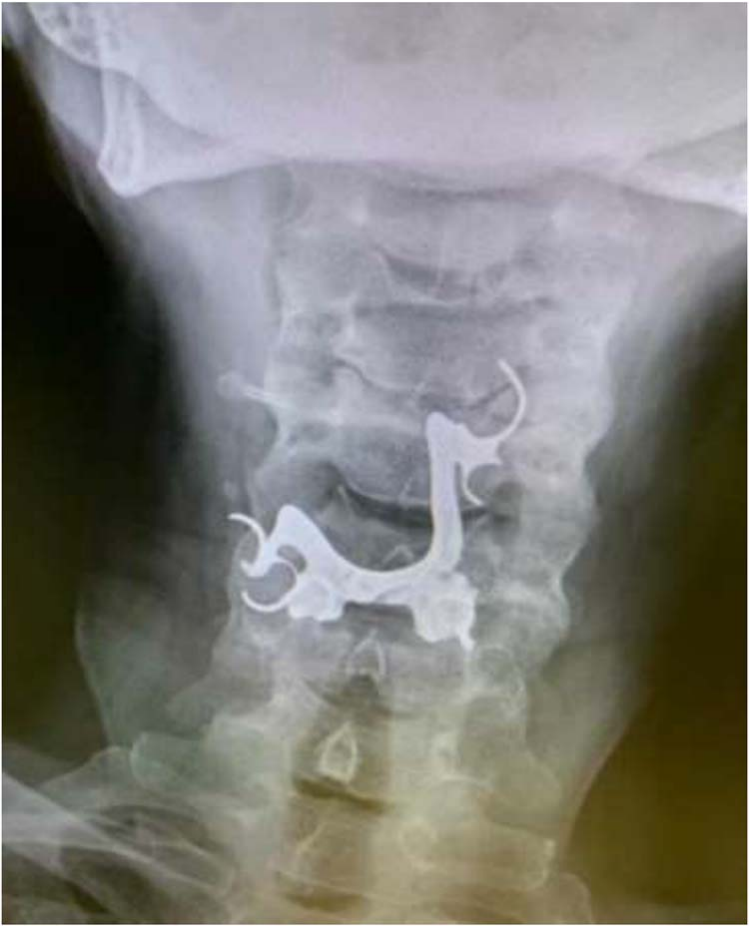
Anterior-posterior neck X-ray showing a radio-opaque object, in keeping with metal dentures.

**Figure 8:**
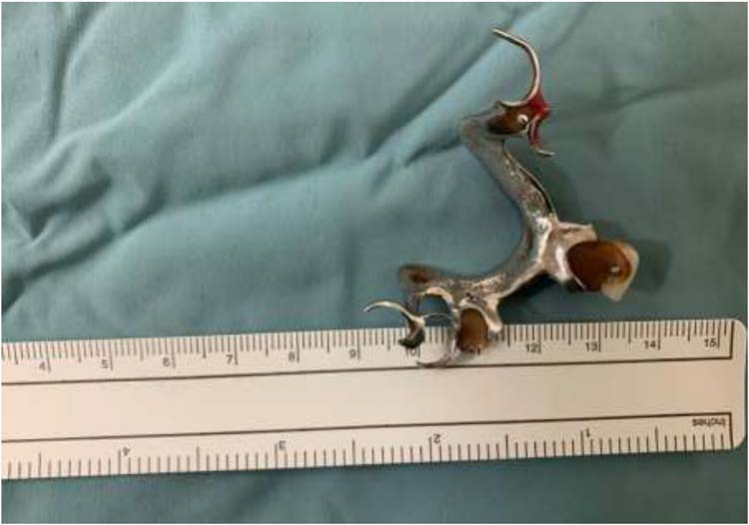
Successful removal of metal dentures.

The final case is of a 75-year-old female who presented to hospital after inhalation of her dentures (Figs 6 and 7). After discussion with the anaesthetic team, apnoeic oxygenation was once again utilized in order to remove the foreign body without risk of dislodgement and to decrease the risk of a potential surgical airway (Fig. 8).

## DISCUSSION

Metallic foreign bodies in the upper aerodigestive tract present a difficult emergency scenario for both otolaryngologists and anaesthetists. Laryngeal foreign bodies can be especially complicated to manage, with an increased risk of airway obstruction and risk of requiring a surgical airway [5]. These often prevent endotracheal intubation due to the object either obstructing the airway or risk of further dislodgement into the airway. Alternative methods of managing the airway and providing a general anaesthetic to a patient with an upper aerodigestive tract foreign body would traditionally include either jet ventilation or spontaneous ventilation. Jet ventilation has many potential complications such as barotrauma, pneumothorax, pneumomediastinum, gastrointestinal insufflation and increased risk of aspiration. Some of these complications, for example aspiration, are more likely to occur in the emergency setting [6]. Spontaneous ventilation may limit the depth of anaesthesia and as a result impede on the success of the procedure [4].

Apnoeic oxygenation using HFNO is a novel technique, which is being increasingly utilized in laryngeal procedures [4]. The technique allows for tubeless anaesthetic and consequently an unobstructed laryngeal field. Using this technique, there is no requirement for bag mask ventilation. HFNO delivers humidified oxygen via nasal cannulae at a rate of greater than 15 L/min compared to a standard nasal oxygenation rate of 6 L/min whilst the patient is awake. After a period of pre-oxygenation using the HFNO device, a general anaesthetic and muscle relaxant are administered using intravenous access, allowing for the surgeon to carry out the procedure. A recent case series evaluating the use of HFNO in microlaryngoscopy procedures, found this technique can allow the surgeon up to 37 min to perform the procedure [4]. In order to allow for ventilation of accumulated carbon dioxide levels, a laryngeal mask airway is typically inserted after completion of the procedure.

A previous randomized controlled trial comparing the use of standard nasal oxygenation with HFNO to allow for apnoeic oxygenation in the removal of foreign bodies during bronchosocopy showed a statistically significant higher level of oxygen saturation and lower level of end tidal carbon dioxide levels in the immediate post-operative period for those in the HFNO group. The use of HFNO has also been shown to have decreased rates of post-operative atelectasis [7].

In light of the novelty of HFNO for apnoeic oxygenation, as well as the associated high stress environment of difficult airway management in the emergency setting, consideration must be given for the anaesthetic experience and familiarity with this technique. HFNO is delivered through specific devices designed to provide humified oxygen at an increased rate. As a result, lack of equipment availability may further hinder the use of this oxygenation method. Increasing training and awareness of this technique will lead to increased accessibility to HFNO in the emergency setting, which may be greatly beneficial in the successful removal of foreign bodies from the upper aerodigestive tract in both the paediatric and adult population.

## CONFLICT OF INTEREST STATEMENT

No conflicts of interest. Informed patient consents obtained.

## FUNDING

None.
